# Supplementary material for: Nepenthes pitcher plant inspired bio-polymer embedded porous surfaces for oil aquaplaning
Source: Sci Rep. 2025 Dec 4;16:1415. doi: 10.1038/s41598-025-31041-x (PMC12796207; doi:10.1038/s41598-025-31041-x)
Supplement: Supplementary file 7 — Supplementary Material 7 [file 41598_2025_31041_MOESM7_ESM.pdf]

Nepenthes pitcher plant inspired bio-polymer embedded porous surfaces for oil aquaplaning

Bethany Orme[1], Alex Jenkins[2], Matthew Unthank[1], Vincent Barrioz[1], Gary Wells[2], Glen McHale[2], Prashant Agrawal[1*]

[1]Faculty of Science and Environment, Northumbria University, Newcastle upon Tyne NE1 8ST, UK.

[2]Institute for Multiscale Thermofluids, School of Engineering, The University of Edinburgh, Kings Building, Edinburgh, EH9 3FB, U.K.

*prashant.agrawal@northumbria.ac.uk

Abstract

The Nepenthes pitcher plant has inspired surfaces employing a liquid barrier that shields the surface from contaminants. However, such liquid-infused oil-repellent surfaces employ toxic fluorocarbons or unstable polar liquids. Here, we develop a bio-friendly oil-repellent surface using a food-grade bio-polymer coating on a nanoporous surface. By mimicking the pitcher plant's nectar lining, the food-grade polymer absorbs water enhancing its stability against drainage and evaporation. The water-based lubricant is non-toxic, compatible with humid environments and replenishes simply by exposure to nebulized water droplets. Our surfaces demonstrate lubrication-induced oil and solid repellence and have substantial transformative potential in applications like oil-water separation, anti-fouling and food packaging.

Introduction

Surface contamination by oil is a concern in many applications, such as the food industry [1, 2], marine environments [3, 4], soil systems [5, 6], wastewater treatment [7] and accretion via atmospheric oil pollution [8]. The low surface energy of many oils means they exhibit high wettability with commonly used surfaces and materials. By understanding the role of surface roughness [9, 10] and chemistry on liquid wettability [11], oleophobic surfaces have been engineered utilizing re-entrant structures [12-14], fluorinated coatings [15, 16] or covalently bonded polymers [17-20].

Texturing surfaces with single and double re-entrant geometries demonstrate excellent super-omniphobicity for liquids with surface tension as low as 10 mN/m. This high liquid repellence is achieved due to a Cassie-Baxter wetting state, where the liquid-solid contact fraction is minimized. However, such physical structures require complex microfabrication procedures and present challenges with mechanical durability and long-term pressure stability [21, 22]. While fabrication of such features historically primarily relied on lithography [23], recent advancements in emulsion-based printing [24] and roll-to-roll

manufacturing [25] have significantly increased process scalability. The sole dependence of liquid repellence on physical structure enables fabrication of metal and polymer-based oleophobic surfaces with enhanced mechanical durability [13]. Additionally, development of armor-like substrates has also improved resistance to scratching and multiple abrasion cycles [26].

Chemical modification of surfaces is another strategy to induce oil repellence, where perfluoroalkyl (PFAS) coatings are commercially the most widely adopted method [27, 28]. In long chain PFAS based coatings, the self-assembly of fluoroalkyl chains and higher density of the $-CF_3$ groups can reduce the surface energy upto 6.7 mN/m [29]. Combined with physical texturing, fluorinated coating surfaces have shown significant pressure stability against drop impact and evaporating liquids [14, 26]. However, low bio-degradability of fluorocarbons, bio-accumulation and their toxicity to the environment and bio-health [30, 31] is an imminent concern that needs stringent global regulations. More recently, covalently bonded alkyl chains have been explored to increase mobility of oil and water droplets [18, 20, 32]. These 'liquid-like' surfaces employ the mobile and dynamically reconfigurable nature of alkyl terminated polymer chains with a low glass transition temperature to reduce droplet pinning on surfaces [33]. While challenges with process scalability of polymer grafting methods and effects of rough surfaces on droplet mobility still remain [11, 32], recent developments in photocurable liquid-like coatings promise potential solutions [34].

Natural designs primarily rely on physical re-entrant geometries to repel oil [12, 35, 36]. While there is no known bio-equivalent of low surface energy materials, the Nepenthes pitcher plant employs a unique solution to repel insects. Predominantly found in tropical climates, the walls of the pitcher can switch their oil repellence by hydration from rainfall or surrounding humidity. This water lubrication repels the oily feet of insects creating both a barrier to the plant surface and a slippery surface. These surfaces have inspired synthetic liquid infused surfaces (LIS) [37-39], where predominantly, oil-infused surfaces have been developed to achieve water-repellence with high droplet mobility [40-42]. Oleophobic liquid-infused surfaces have been proposed using toxic fluorocarbons as lubricants, but these surfaces pose a higher health risk to the environment through lubricant depletion [38, 43, 44]. Some edible oil infused surfaces also provide sustainable bio-friendly alternatives for achieving selective oil repellence [45, 46]. While a small number of studies have proposed water infused porous surfaces [47, 48], evaporation and drainage stability of the water lubricant remains an unsolved challenge [7, 49].

In this work, we replicate a Nepenthes pitcher plant's mechanism of trapping insects, to develop bio-friendly oil-repellent surfaces. Analogous to the nectar lining in the pitcher plant, which adsorbs and holds rainwater on its surface, we use a common food-grade polymer to develop a stable, non-toxic and environmentally friendly water-based lubricated surface to repel oil and solid contaminants. The nanoporous texture of the surface stabilises the lubricant against drainage while the bio-polymer provides stability against evaporation. Additionally, similar to the pitcher plant, the lubricant and its oil-repellent properties can be replenished just by soaking in water. Our bio-friendly, oil-repellent surfaces offer a sustainable, transformative alternative for applications like oil-water separation and food packaging.

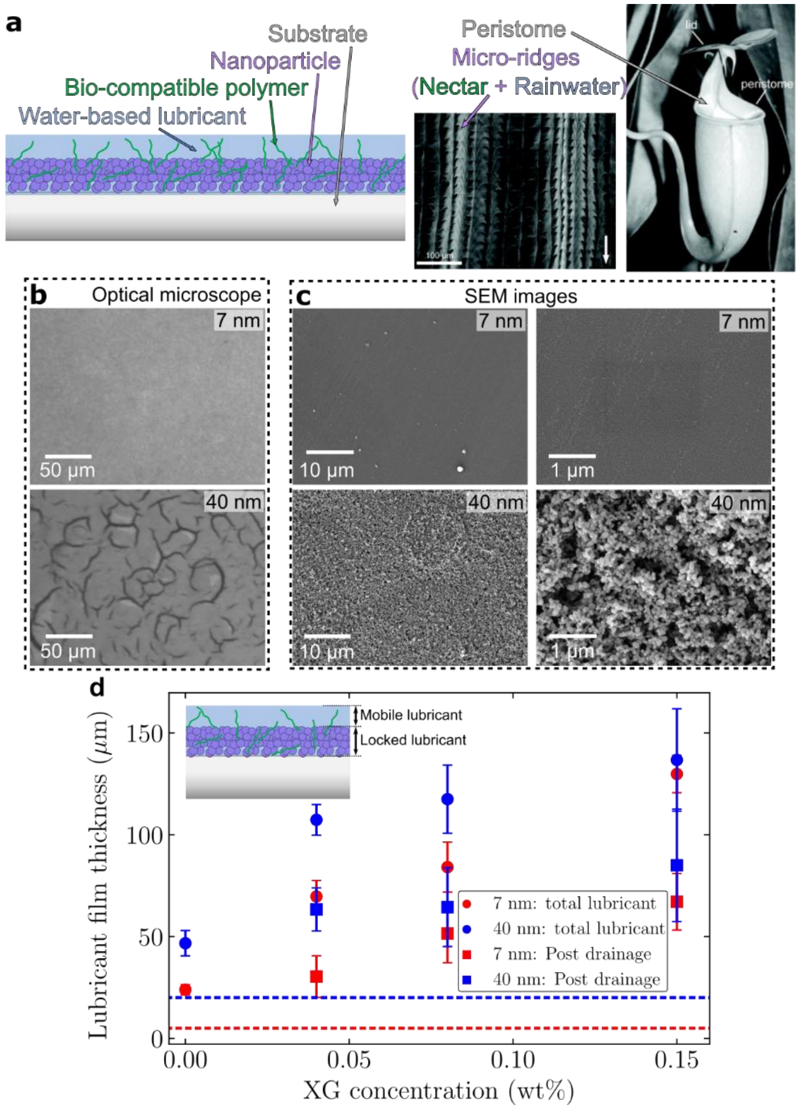

Figure 1. Design of a synthetic Nepenthes pitcher plant. **a**, Concept of our water-based liquid infused surface inspired from the Nepenthes pitcher plant (image taken from [50], Copyright (2004) National Academy of Sciences, U.S.A.): A super-hydrophilic silica nanoparticle coating, analogous to the peristome of the pitcher plant, holds the water-xanthan lubricant, where xanthan stabilizes water similar to nectar in a pitcher plant. **b**, Bright field microscope images. **c**, SEM images of the nanoporous surface for two different particle sizes: with average diameter 7 nm and 40 nm; the average thickness of the nanoporous coating is 5.0 μm ± 1.0 μm and 20.3 μm ± 2.6 μm, respectively (Figure S1). **d**, Lubricant thickness for different xanthan (XG) concentrations on the two nanoporous surfaces (7 nm and 40 nm). Total lubricant thickness (circles) is obtained just after imbibing the lubricant. The mobile lubricant drains via gravity resulting in a gravity-stabilized lubricant thickness (squares). The dashed red and blue lines represent the thickness of the nanoporous coating for the 7 nm and 40 nm particles, respectively.

Results

Concept

The peristome of a Nepenthes pitcher plant combines a ridge-like micro texture along with secretion of a sugary nectar to ensure a continuously wetted and lubricated surface in humid tropical environments, which repels oil on the insect's adhesive pads. The micro-roughness ensures a completely wetted surface while the hygroscopic nectar ensures a continuous presence of water over the surface. In developing a synthetic version of a Nepenthes pitcher plant, we replace each of these components with a synthetic equivalent (Figure 1a). The micro-roughness of the peristome is replicated using a super hydrophilic nanoporous coating (Figure 1b, c), while a bio-compatible food-grade polymer, xanthan gum, is used as an equivalent for the nectar. Xanthan is dissolved in water in different concentrations and the solution is used to create an environmentally friendly and stable lubricant, which is infused in the nanoporous coating.

Lubricant stability

A fundamental barrier to realize water lubricated surfaces is to ensure stable locking of the lubricant on the surface against drainage and evaporation. We achieve this through a combination of the nanoporous coating and xanthan. The addition of Xanthan primarily enhances the stability of the lubricant against evaporation (Figure 2a). For instance, by adding just 0.04 wt% xanthan the evaporation time increases from less than 15 minutes for de-ionized water infused surfaces to around 81 minutes (Figure 2a). Further increasing the xanthan concentration to 0.15 wt% increases the evaporation time more than 700% to almost 115 minutes.

The decrease in the evaporation rate by the addition of Xanthan is attributed to the availability of water for evaporation during the drying process. As seen in Figure 2b, c, while drying, sugars form a layer on the particles, which shields the dissolved water in the bulk from evaporation and reduces the rate of evaporation. This concept of shielding water is used in the agriculture industry, where water soluble polymers (synthetic and natural) are used to treat soil particles for enhanced water retention [51-53]. Additionally, sugars reduce the water activity of a solution by binding with water molecules, which reduces the availability of water for evaporation. This concept of increasing water retention by adding sugars is used widely to limit bacterial growth in food preservation [54, 55]. Therefore, increasing Xanthan concentrations beyond those used on our surfaces can significantly extend evaporation rates further (Figure S3 in supplementary information).

The nanoparticle coating is used as a reservoir for the xanthan-water lubricant, where a part of the lubricant is locked and held stably due to the super-hydrophilicity of the nanoparticles (depicted in Figure 1d). The remaining excess lubricant is mobile and is prone to drainage (or leaching) over time due to gravity or shear in real environments. The concentration of xanthan in the lubricant affects the amount of the excess (mobile)

lubricant left on the surface. An increase in the xanthan concentration increases the viscosity of the lubricant (Figure S2) and, therefore, reduces the movement of the mobile lubricant on the surface. As a result, after about 60 minutes of drainage, the thickness of the lubricant film is higher for a higher xanthan concentration (Figure 1d).

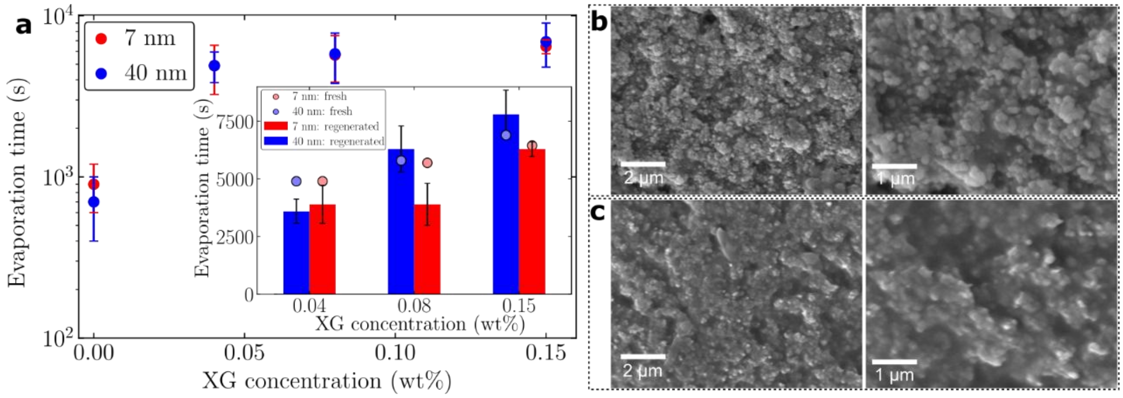

Figure 2. Effect of xanthan on lubricant evaporation. **a,** Evaporation time of the freshly lubricant impregnated nanoporous surfaces for different xanthan concentrations; 0 wt% implies pure water. The inset shows a comparison of the evaporation times of the freshly lubricant impregnated surfaces (circles, depicting the average time) with the regenerated lubricant impregnated surfaces. The regenerated lubricant is obtained by soaking the dry polymer coated nanoporous surfaces in water, which allows the polymer to re-dissolve in the water. **b,** SEM images of 40 nm surfaces dried after imbibing a xanthan-water solution with 0.04 wt% xanthan. After drying the xanthan gum coats the particle and assists in retaining water. c, SEM images of 40 nm surfaces dried after imbibing a xanthan-water solution with 0.15 wt% xanthan.

Rehydration and regeneration

Nature relies on replenishment via biological regeneration to retain the wettability properties of surfaces. A key aspect of water retention by the pitcher plant is the ability of the peristome to regenerate the nectar and re-hydrate periodically by water absorption due to condensation from ambient humidity and rainfall [56, 57]. Our synthetic surfaces demonstrate a similar rehydration capability while retaining xanthan on the surface. After the water has evaporated from the lubricant, the xanthan polymer is left behind and coats the nanoparticle surface (Figure 2b, c). When water is re-infused in the surface and left for 24 hours in high humidity conditions, the polymer re-dissolves in water and regains its original lubricant characteristics. This regeneration of the lubricant is characterized by measuring the evaporation rate, where the regenerated lubricant surfaces show a similar evaporation time scale as the freshly water-xanthan lubricant impregnated surfaces (Figure 2a). The high humidity condition is critical for a complete dissolution of the polymer in water to ensure a higher stability against evaporation. When a dry polymer coated surface is infused (or rehydrated) with water in ambient conditions, it leads to incomplete dissolution of the polymer in water, which is indicated by an evaporation rate similar to pure water lubricated surfaces (Table S3 in supplementary information). The xanthan polymer can leach off from the surface over time due to drainage by water or shear in real

environments, which can reduce the water retention capability of the surface. However, xanthan can be reintroduced on the surface via re-infusion with a water-xanthan solution. Importantly, due to the bio-friendly nature of the lubricant, leaching of the lubricant does not pose a contamination risk to the environment.

Preferential wetting to water

In their dry state, the nanoporous nature of coatings renders them super-amphiphilic [58], which allows the complete spreading of both water and oil-based lubricants. Therefore, for the water-xanthan lubricated surface to be oil-repellant, the lubricant should preferentially wet the surface over oil. The condition for preferential wetting of the water-based lubricant can be given as (Supplementary information) [59]: $S_{so(a)} - S_{sw(a)} \leq \gamma_{wa} - \gamma_{oa} - \gamma_{ow}$, where, $S_{so(a)}$ and $S_{sw(a)}$ are the spreading coefficient of oil and water on solid in the presence of air, respectively, and, γ_{ow}, γ_{oa} and γ_{wa} are the surface tension of the oil-water interface, oil-air interface and water-air interface. Plugging in the values of the different surface tensions, we obtain $S_{so(a)} - S_{sw(a)} \leq 14.2$.

Therefore, the preferential wetting of water-based lubricants can be induced by altering the relatives spreading coefficients of oil and water on the surface in air. For example, surfaces like stainless steel and brass display a high advancing and receding contact angle for water-xanthan solutions (Table S4), i.e. $S_{sw(a)} < 0$. On these substrates, an oil drop completely wets the surface, i.e., $S_{so(a)} \geq 0$, which does not satisfy the condition $S_{so(a)} - S_{sw(a)} \leq 14.2$. Therefore, when a water-xanthan droplet is placed on an untreated oil infused substrate (Figure 3a), the oil preferentially wets the substrate. However, the high sliding angle of these water-xanthan droplets (approximately 28 ± 6°) indicates that the droplets are in an emergent-impregnated state on the substrate (Figure 3d, g), rather than complete oil encapsulation ($S_{os(w)} \geq 0$) [59]. When the surfaces are treated with a hydrophilic coating (Figure 3b, static contact angle of water in air: $\theta_w \approx 10°$), the spreading coefficient $S_{sw(a)}$ increases such that the water-xanthan solution displaces the oil and preferentially wets the surface (Figure 3e, h and movie S6), implying that condition $S_{so(a)} - S_{sw(a)} \leq 14.2$ is satisfied. The preferential wetting of water-based lubricants is also observed on the nanoparticle coated substrates (Figure 3c, f). The nanoparticle coating additionally ensures that the lubricant remains locked on the surface and acts as a reservoir. When a drop of the water-xanthan is deposited on an oil infused surface, the lubricant droplet displaces the oil and impregnates the nanoporous coating (Figure 3f and movie S1 and S6). Furthermore, on rinsing the oil-infused surface with the xanthan solution, oil is completely displaced from the surface (movie S1). The complete removal of the infused oil is also verified using confocal microscopy (Figure S7 in supplementary information).

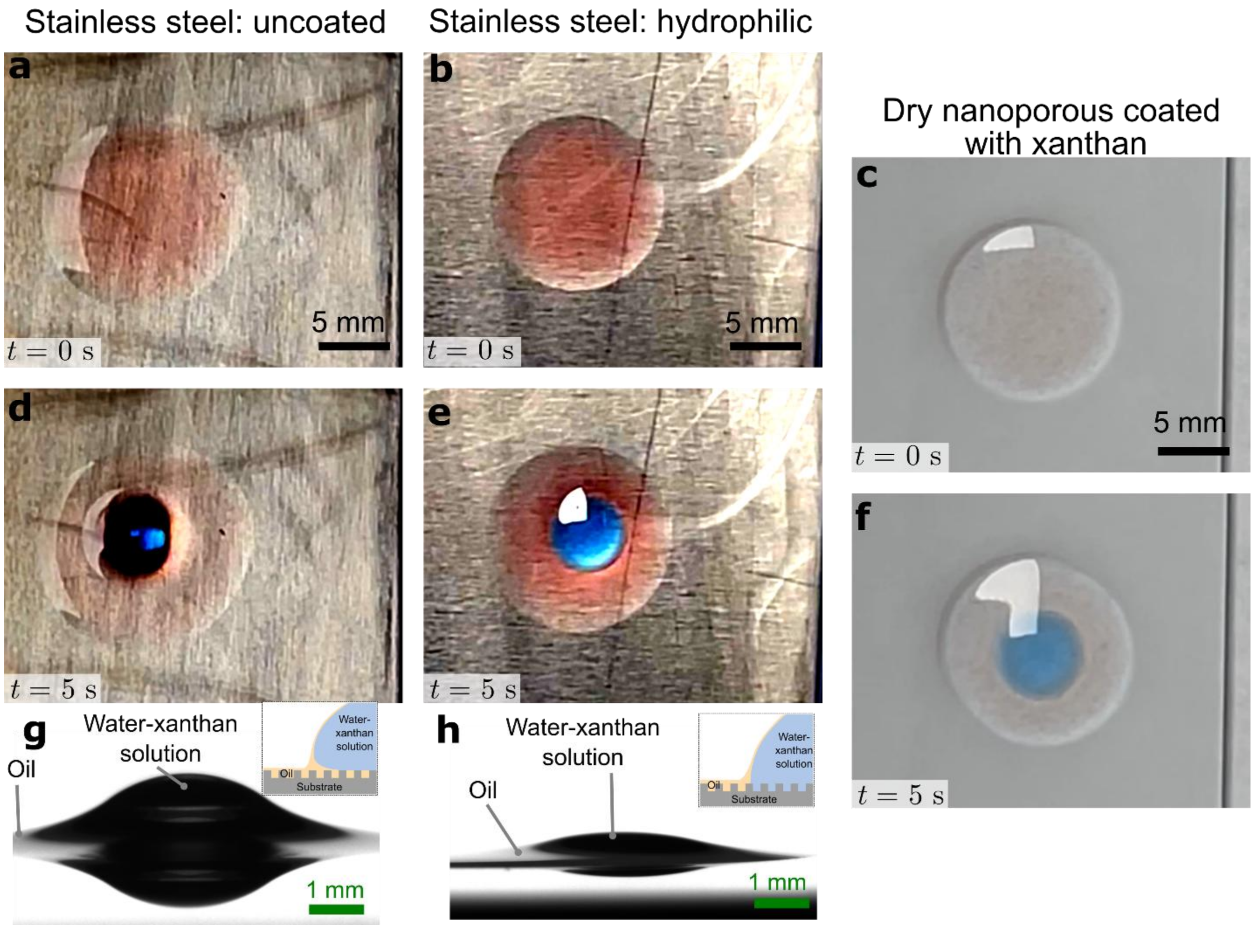

Figure 3. Preferential wetting by water-based lubricants. 10 µl dyed silicone oil (red) droplet spreading as a film on: **a,** untreated stainless-steel substrate; **b,** hydrophilic-treated stainless steel surface (using P100) and **c,** dry xanthan coated nanoporous glass substrate. A 10 µl drop of 0.08 wt% xanthan-water solution (mixed with blue colored food-grade dye) is placed on the oil film on the different substrates and observed after 5 seconds: **d,** water-xanthan solution does not completely impregnate the substrate; **e,** water-xanthan solution displaces the oil and spreads on the steel surface, evident with a relatively larger spread radius compared to **d**; **f**, water-xanthan solution displaces the oil and imbibes in the nanoporous coating; **g**, side view image of the water-xanthan solution on oil in **b**, indicating an emerged impregnated wetting state; **h**, side view image of the water xanthan solution on oil in **e**, indicating an impaled wetting state. Corresponding video is provided in Movie S6.

Oil repellence

Similar to the oily feet of insects on a pitcher plant, the water-xanthan lubricant ensures oil droplets remain separated from the nanoporous surface. While the water-xanthan lubricant impregnated surfaces are naturally oil repellent, the nanoporous structure can either be completely encapsulated by the lubricant or it can remain in a partially emerged-impregnated state (Figure S6 in Supplementary information). The key distinguishing

feature between the two wetting states is that oil remains completely separated from the nanoporous structure in the encapsulated state, while it encounters pinning in the emerged-impregnated state [59]. Due to the oleophilic nature of the nanoporous surface, it is expected that the pinning forces will be significantly high in the emergent-impregnated state[59]. The sunflower oil and rapeseed oil droplets form a lens-like configuration, while the sesame and silicone oil droplets spread rapidly into a thin-film due to the low surface energy of their water-oil interfaces [60] (Movie S2), which complicates accurate measurement of sliding angles. The sunflower and rapeseed oil (Figure 4a, b and movie S3) droplets are seen to slide on the surface due to gravity with just a 5° tilt, indicating that the oils remain completely separated from the surface, i.e., the nanoporous structure is completely encapsulated by the lubricant (depicted in Figure S6). The sesame and silicone oil droplets spread over the surface (Figure 4c, d) and can be removed via rinsing with the lubricant solution (Figure 4e and Movie S4) or with water.

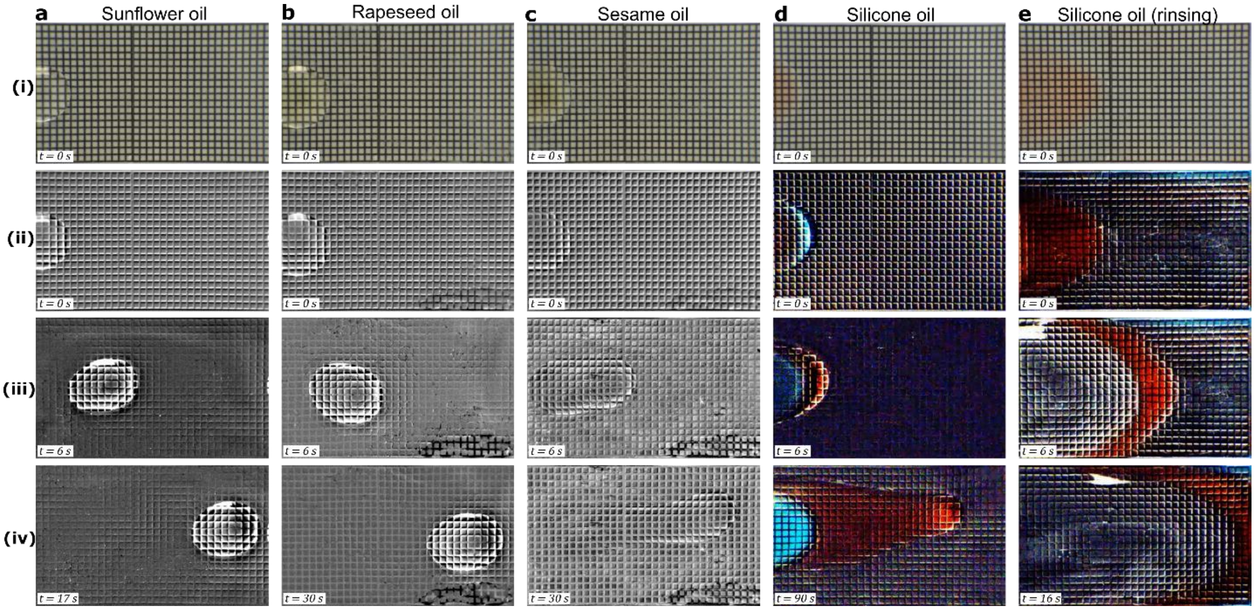

Figure 4. Oil repellence on the water-xanthan lubricated surface. A 13 µl droplet of different oils is deposited on a 0.04 wt% xanthan-water lubricated nanoporous surface (particle diameter 7 nm) tilted at 5°. **a,** Sunflower oil. **b,** Rapeseed. **c,** Sesame. **d,** dyed 20cSt Silicone oil. **e,** Sequence of images showing removal of dyed 20 cSt silicone oil via rinsing with xanthan-water solution 0.04 wt%. A unit cell in the grid is 1 mm x 1 mm. Images in row (i) show the raw image just after drop deposition (before substrate tilt). Images in row (ii) show the digitally processed images from (i). Images in row (iii) and (iv) are digitally processed at different times after the substrate is tilted. Corresponding video is available in Movie S3 and S4.

Solid contaminant removal

The presence of a continuous lubricant layer between external solid contaminants and the surface ensures protection of the underlying surface. The lubricant also induces an easy removal of different types of solid and semi-solid (like tomato ketchup) contaminants. Glass particles (10 µm), regular sand and hydrophobic sand deposited on

the lubricated surface are easily removed under gravity because of the mobile lubricant layer and can be easily removed from the surface by rinsing with water (Movie S5). Additionally, a droplet containing a dispersion of polystyrene particles (10 μm) was deposited on the surface to simulate the interaction with rainwater. Though the particles spread immediately on the surface (due to the water base), the particles are mobile on the lubricant layer and can be removed by tilting the surface or rinsing with DI water (Figure S9).

Optical transparency

The use of a water-based lubricant imbibed in a silica nanoparticle coated surface allows use of these oil repellant surfaces in applications where optical transparency is required. In the visible range (380 nm – 700 nm), almost 13% of the light transmits diffusively through the dry 7nm nanoparticle coated surfaces, compared to uncoated glass sample (Figure 5a). For the surface coated with 40nm nanoparticles, diffusive light transmission increases to 35%. The total light transmission is also lower for the 40nm coated surface (Figure 5b). However, the addition of the lubricant decreases the diffusive light transmittance and increases the total light transmission through the substrates (Figure 5a, b), irrespective of the particle sizes. Additionally, for the considered weight percentages, xanthan concentration in the lubricated film does not have a noticeable effect on the light transmittance through the lubricated sample. Light transmission also increases by exposing the dried polymer coated surface with a nebulized spray of water (Figure 5c). However, the water evaporates within 500 seconds from the surface because the polymer is not hydrated and dissolved in the water completely (Table S3).

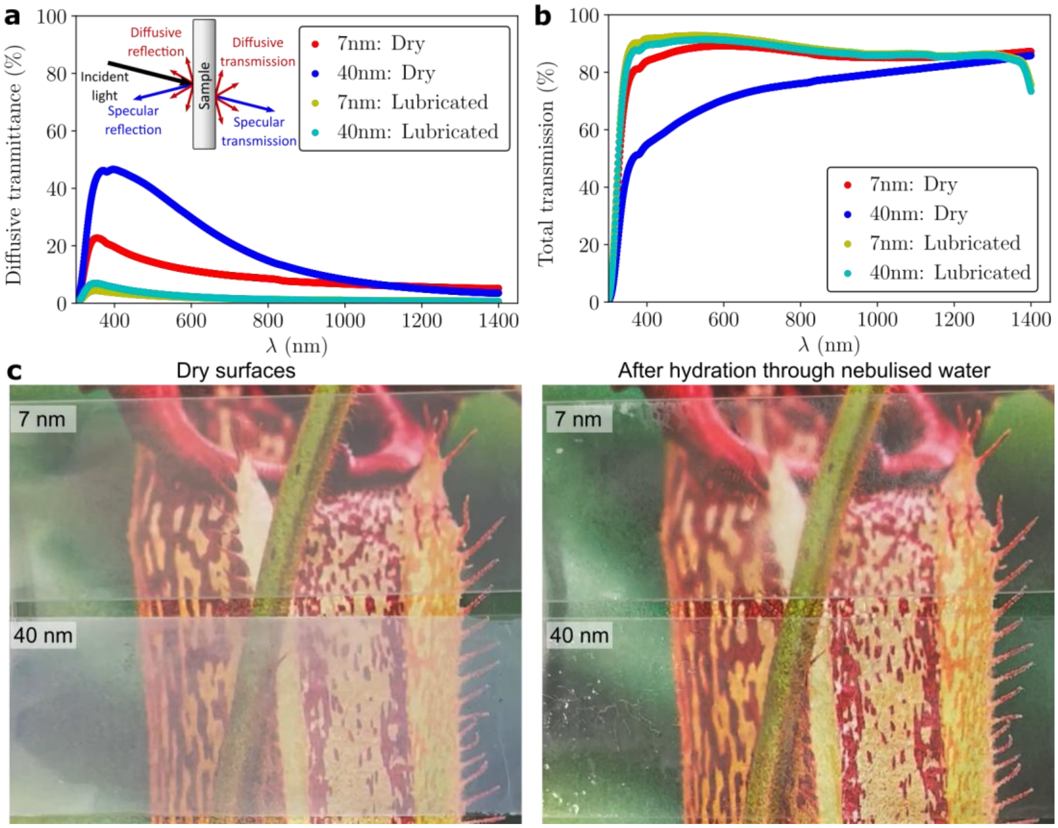

Figure 5. Characterization of optical transparency. **a,** Diffusion of light through the dry and lubricated surfaces for the 7nm and 40nm particle sizes. Inset shows a depiction of different components of light reflection and transmission. **b,** Transmission of light through the dry and lubricated surfaces for the 7nm and 40nm particle sizes. The transmittance values are in reference to an uncoated glass slide. **c,** Restoration of optical transparency of the surface after rehydration through a nebulized water spray (Photo credits of the Pitcher plant: Geoff McKay, https://flickr.com/photos/129472387@N07/49360096312, Copyright license CC-by-2.0).

Conclusion

In summary, through a like-for-like replacement of the different components that allows a Nepenthes pitcher plant to trap insects, we have created a water-based lubricant infused, oil repellent, porous surface. Adapting the practices of the food and agriculture industries to control water in food and soils, respectively, our key observation is that the addition of a small amount of the bio-polymer xanthan-gum (replicating the nectar of a pitcher plant) significantly reduces the evaporation rate and drainage of the water-based lubricant. A constant layer of a water lubricant on the surface inhibits contamination from oil and solid particulates, which can be removed via gravity or rinsing with water. Though the water in the lubricant still undergoes evaporation, the lubricant, and the surface's oil repellence, can be regenerated either by adding more lubricating liquid or by soaking the surface in water. The concept of coating surfaces with food grade polymers instead of the toxic 'forever chemicals' per- and poly- fluoroalkyl substances offers a powerful bio-friendly and sustainable strategy to achieve oil repellence for applications in oil-water separation, optical coatings and food packaging.

Methods

Substrate cleaning

Plain soda lime glass microscope slides (Fisherbrand) of dimensions 75 mm x 25 mm were cleaned by placing the substrates vertically into a beaker filled with a 2 wt% Decon90 solution and sonicated for 15 mins at 30 °C. This sonication step removes all organic contaminants from the substrate. The substrates sonicated in Decon90 were then rinsed with de-ionised (DI) water (resistivity 18 MΩ-cm) and again sonicated vertically in DI water for 15 mins at 30 °C to remove any Decon90 remnants on the substrate. The substrates were then individually and sequentially rinsed with acetone, iso-propanol (IPA) and DI water before being fully dried with compressed air.

Nanoparticle preparation

Two hydrophilic fumed silica particles of different sizes were used to prepare the nanoporous coating: approximately 7 nm and 40 nm (Aerosil 300 and Aerosil OX50, respectively, supplied by Evonik Industries). 2 g of each particle diameter were weighed into two separate beakers and dispersed into 40 g of IPA to create a 5 wt% slurry [61]. To ensure full dispersion, a tip ultra-sonicator (Vibra Cell CV334) is placed into the beaker and sealed with parafilm to prevent evaporation and pulsed over 3 seconds for a total of 20 minutes. The sonication process heats the mixture, therefore, to maintain the temperature of the mixture between 20 and 25 °C, the beaker was placed in an ice bath, while continuously monitoring the temperature using a thermocouple. After the tip sonication, the dispersed particle mixture is stored in sealed containers. Although the fumed silica particles are intrinsically hydrophilic and can, theoretically, form a superhydrophilic nanoporous coating upon spraying, our initial experiments showed that when these nanoparticles were sprayed directly on the substrate, the particles were removed

easily by the imbibing lubricant. Therefore, 10 g of the prepared particle mixture was added to 10 g of P100 (an IPA based hydrophilic coating provided by Jonnin) and then mixed in an ultrasonic bath (Fisherbrand FB15053) for 20 minutes at a temperature of 30 °C. P100 retains the hydrophilicity of the nanoparticles and also assists in adhering the particles on the substrate, even after liquid imbibition.

Nanoparticle spray coating

The feed hopper of a spray gun (Sparmax GP50) was filled with 0.5 mL of the nanoparticle mixture and was attached to a compressor (AS196) set to 1 bar. The cleaned glass slides were given an IPA rinse and allowed to dry in a fume hood. The mixture was then sprayed on these slides at a distance of approximately 20 cm, ensuring the entire surface of the slides was covered. The samples were left in the fume hood for 60 seconds to ensure all solvents had evaporated before another layer of coating was performed. The process was repeated 5 times to obtain a dry coating.

Nanoparticle surface characterization

An optical microscope (Nikon Eclipse LV100) and a scanning electron microscope (Tescan MIRA3) were used to image the nanoparticle coated substrates. Cross-sectional SEM images were also taken to measure the particle coating thickness. For the SEM images, samples were snapped over a thick tungsten wire to create a natural break in the particle coating.

Lubricant fabrication

A biopolymer, xanthan gum (derived from Xanthomonas Campestris, Sigma Aldrich) was hydrated into DI water to create three suspensions of different xanthan concentrations: 0.04 wt% (400 ppm), 0.08 wt% (800 ppm) and 0.15 wt% (1500 ppm). The fabrication process for preparing xanthan-water solutions has been adopted from the work of Jaishankar et al [62]. To prepare these solutions, 0.04 g, 0.08 g, and 0.15 g of xanthan gum was weighed (using a Denver Instruments balance TP-214) and added to DI water to prepare a solution with mass 100 g. These xanthan-water mixtures were stirred in a sealed container using a magnetic stirrer at 300 rpm for 24 hours at room temperature (20 to 25°C) to stretch and disperse the polymers uniformly in the water. After stirring, the solutions are stored at 4 °C for 12 hours to complete the polymer hydration.

Lubricant rheology

The viscosity of the solutions was measured using a rotational rheometer equipped with a 1°/50 mm cone and plate geometry at 25 °C (Kinexus Lab+). A shear rate ramp up from 0.01 s-1 to 100 s-1 and a ramp down from 100 s-1 to 0.01 s-1 was performed on all the samples.

Lubricant infusion and thickness measurement

Each of the xanthan-water solutions were added to the nanoparticle coated surfaces using a 1 mL syringe to cover the entire substrate with the liquid. The samples were then tilted by 90° to allow the excess liquid to run off the surface.

The lubricant thickness was calculated by measuring the mass of the infused lubricant. The mass of the nanoparticle coated substrates was measured prior to and after adding the lubricant, as discussed above. The difference of these measurements provides the lubricant mass. The lubricant thickness is then calculated from the volume of the lubricant and the substrate area. The total lubricant comprises of the lubricant that is locked in the nanoporous coating and a mobile lubricant that is prone to drainage over time. The thickness of this mobile lubricating layer is obtained by subtracting the thickness of the nanoporous coating.

Lubricant stability test: drainage and evaporation

Lubricated substrates prepared using the above method were hung at 90° for 60 minutes in a high relative humidity sealed chamber (RH > 90%) to limit the lubricant evaporation. The lubricant drained from the samples was collected in a collection tray at the base of the samples. The mass of the drained lubricant was weighed to obtain the mass of the remaining lubricant on the surface.

Lubricated samples were allowed to evaporate in ambient conditions (Average environmental temperature: 23.9 ± 4.1 °C and relative humidity: 40.7 ± 4.5%) and images were taken every five minutes. The evaporation time was defined by the time it took for the entire surface to become dry.

Effect of water activity test: Droplet evaporation

To investigate the drying dynamics of the water-xanthan solutions, initially droplets of 4 μL were deposited on Slippery Omniphobic Covalently Attached Liquid-like (SOCAL) surfaces prepared as in the method by Armstrong et al [63]. The surfaces were placed in a bespoke enclosed environmental chamber. The humidity of the surrounding air was controlled at 60±2% using an in-house manufactured humidity controller and the temperature was held at 25±0.2 °C using a Thorlabs temperature-controlled stage (ThorLabs, PTC1/M). The xanthan concentrations used in the experiments were, 0.125 wt% (1250 ppm), 0.25 wt% (2500 ppm), 0.5 wt% (5000 ppm) and 1 wt% (10000 ppm) solutions. The evaporating droplets were imaged using a Raspberry Pi HQ cam with a C-Mount Microscope lens. Using these images, the profile of the droplet was extracted using a bespoke open-source drop shape analysis tool (pyDSA) and the volume of the droplet was determined by assuming axial symmetry. The data for 1 wt% droplets proved inaccurate and is not analyzed because the extreme viscosity of the solution led to asymmetric droplets which led to inaccurate determination of the volume.

Effect of water activity test: Film evaporation

To measure the rate of mass loss for a thin film of water-xanthan lubricant, 7 nm nanoparticle coated glass substrates were spin coated on a Laurell Spin Coater (WS-650Mz-23NPPB) with solutions of different concentrations. Each sample was first spun at 250 rpm for 30 seconds and then a subsequent spin, which was calibrated to achieve the same layer thickness irrespective of the xanthan concentration. These secondary spin speeds were: 2500 rpm for 30 seconds for 1 wt% solution, 1000 rpm for 30 seconds for 0.125 wt% solution and 1000 rpm for 10 seconds for water (Ultra Pure DI, Simga Aldrich). The samples were then immediately transferred to an accurate mass balance (AdamDU analytical scale, accuracy of ±0.1 mg) and the mass measured at 10 second intervals using the AdamDU software. During the evaporation of the thin film the humidity inside the mass balance enclosure was controlled at 60±2% using the same humidity controller as the droplet experiments.

Lubricant regeneration via rehydration

Re-hydration of dry polymer coated samples (i.e., samples that had previously been infused with xanthan-water solutions of varying concentrations and dried in ambient conditions) was investigated using two methods.

In the first method dry polymer coated samples were rehydrated with DI water by applying a film of water (volume between 0.75 mL and 0.5 mL for samples originally lubricated with xanthan concentrations 0.04 wt% and 0.15 wt%, respectively). The samples were sealed for 24 hours to replicate the hydration stage of polymers during the fabrication of the lubricants. After the 24-hour rehydration time had elapsed, the samples were allowed to evaporate in ambient laboratory conditions following the same experimental procedure described in the section Lubricant stability: drainage and evaporation.

A second method was explored by exposing the dry polymer coated surface to a water spray from a nebulizer. The dried sample that was previously lubricated with 0.15 wt% xanthan solution was exposed to nebulized water (generated using Omron Nebulizer NE-U780 UltraAir Pro) for five minutes. After this, the samples were weighed to estimate the thickness of the infused lubricant via its mass, as per the procedure described in the section Lubricant infusion and thickness measurement. These nebulizer rehydrated surfaces were immediately placed in ambient conditions to evaporate (not allowing a 24-hour rehydration step as in the first method), which provides a qualitative measure of the extent to which the polymer has dissolved in the nebulized water. The evaporation times provide an indication of whether the water-xanthan lubricant has regenerated and regained its stability characteristics as in a freshly lubricated sample.

Optical transparency measurement

The optical properties of the lubricated substrates were measured using a UV-Vis spectrometer (Shimadzu UV-2600 plus spectrophotometer), where the substrates were exposed to light wavelengths between 300 nm and 1400 nm. The spectrometer is equipped with an integrating sphere with multiple sample placement configurations allowing total, specular and diffused transmittance to be measured, effectively enabling the haze to be calculated. The haze is a measure of the light scattered diffusively with respect to the total light transmitted through a substrate. Therefore, Haze is calculated as a ratio of the diffused transmittance to the total transmittance. The transmittance values are obtained against a reference sample, which is an uncoated soda lime glass slide.

Dyed oil preparation

Silicone oil is dyed with 0.01 wt% Nile Red, which is a fluorescent lyophilic dye to visualize the oil optically and in the confocal microscope; the dye has an excitation wavelength of 530 nm and emission wavelength of 635 nm. The solid Nile Red dye was first dissolved in toluene to create a 0.1 wt% Nile Red/Toluene solution. The dyed Nile Red was then added to the silicone oil and the toluene allowed to evaporate from the dyed silicone oil overnight in a fume hood before being used on samples.

Oil sliding experiments

To measure the sliding properties of the surfaces, the substrate was placed on a tilting stage (Thorlabs RP03), which is levelled using a spirit level and a 4 μL droplet is placed on the substrate. The stage is then tilted manually to 5° to allow the oil droplets to slide.

Confocal microscope

An inverted Leica DMi8 microscope with a FTIR cube is used to view the fluorescence of the sample when coated, when lubricated and in the presence of the dyed oil. The surfaces were placed on the microscope and a z-scan performed with a step size of 1 μm.

Preferential oil wetting test

A 4 μL drop of dyed silicone oil is infused in the dry nanoporous substrate. The oil infused surface is then rinsed with 2 mL of 0.04 wt% xanthan solution.

Oil penetration over time test

A substrate lubricated with 0.04 wt% xanthan is used and a 4 μL drop of silicone oil is placed on the lubricated sample. The sample was airtight sealed in a petri dish to avoid lubricant evaporation for 2 hours. Post this process, the sample was imaged at the same position.

Oil removal by rinsing test

Samples are rinsed with xanthan solutions via gravity by adding 1-2 mL of the relevant xanthan solution to the samples to remove the buoyant or infused oils.

Author Contributions

B.V.O and P.A. conceptualized the idea, B.V.O, M.U., P.A., G.G.W. developed the fabrication and characterization process, B.V.O. conducted the experiments, A.J. conducted confocal microscopy measurements and evaporation tests, V.B. conducted optical spectroscopy measurements. All authors contributed to the data analysis, writing and review of the manuscript.

Funding Sources

The authors acknowledge funding support from Northumbria University, U.K.

Competing interests

The author(s) declare no competing interests.

Data availability

The datasets generated during and/or analyzed during the current study are available from the corresponding author on reasonable request.

References

[1] S. Zouaghi *et al.*, Antifouling Biomimetic Liquid-Infused Stainless Steel: Application to Dairy Industrial Processing, *ACS Appl Mater Interfaces,* 9, 31, 26565-26573 (2017).

[2] K. Yi, S. Fu, H. Zhang, H. Zhang, Y. Wang, and Y. Huang, Cellulose nanofibrils/polydimethylsiloxane double-layer coating for fabrication of high barrier and excellent water- and oil-resistance paper, *Progress in Organic Coatings,* 172, (2022).

[3] P. Wang, D. Zhang, S. Sun, T. Li, and Y. Sun, Fabrication of Slippery Lubricant-Infused Porous Surface with High Underwater Transparency for the Control of Marine Biofouling, *ACS Appl Mater Interfaces,* 9, 1, 972-982 (2017).

[4] X. Li, Q. Wang, L. Lei, Z. Shi, and M. Zhang, Amphiphobic concrete with good oil stain resistance and anti-corrosion properties used in marine environment, *Construction and Building Materials,* 299, (2021).

[5] G. Lassalle, A. Credoz, R. Hedacq, S. Fabre, D. Dubucq, and A. Elger, Assessing Soil Contamination Due to Oil and Gas Production Using Vegetation Hyperspectral Reflectance, *Environ Sci Technol,* 52, 4, 1756-1764 (2018).

[6] C. Streche, D. M. Cocarta, I. A. Istrate, and A. A. Badea, Decontamination of Petroleum-Contaminated Soils Using The Electrochemical Technique: Remediation Degree and Energy Consumption, *Sci Rep,* 8, 1, 3272 (2018).

[7] T. J. Ko *et al.*, Direct recovery of spilled oil using hierarchically porous oil scoop with capillary-induced anti-oil-fouling, *J Hazard Mater,* 410, 124549 (2021).

[8] Y. Zhang *et al.*, The Fabrication of Oleophobic Coating and Its Application in Particulates Filtration, *Coatings,* 12, 7, (2022).

[9] J. Lou *et al.*, Polygonal non-wetting droplets on microtextured surfaces, *Nat Commun,* 13, 1, 2685 (2022).
[10] V. Liimatainen *et al.*, Mapping microscale wetting variations on biological and synthetic water-repellent surfaces, *Nat Commun,* 8, 1, 1798 (2017).
[11] A. Dhyani, J. Wang, A. K. Halvey, B. Macdonald, G. Mehta, and A. Tuteja, Design and applications of surfaces that control the accretion of matter, *Science,* 373, 6552, (2021).
[12] A. Tuteja, W. Choi, J. M. Mabry, G. H. McKinley, and R. E. Cohen, Designing superoleophobic surfaces, *Science,* 318, 1618–1623 (2007).
[13] T. Liu and C. J. Kim, Turning a surface superrepellent even to completely wetting liquids, *Science,* 346, 1096–1100 (2014).
[14] G. T. Yun *et al.*, Springtail-inspired superomniphobic surface withextreme pressure resistance, *Science Advances,* 4, (2018).
[15] X. Deng, L. Mammen, H. J. Butt, and D. Vollmer, Candle soot as a template for a transparent robust superamphiphobic coating, *Science,* 335, 6064, 67-70 (2012).
[16] M. J. Hoque *et al.*, Ultra-resilient multi-layer fluorinated diamond like carbon hydrophobic surfaces, *Nature Communications,* 14, 1, 4902 (2023).
[17] M. Liu, Z. Wang, P. Liu, Z. Wang, H. Yao, and X. Yao, Supramolecular silicone coating capable of strong substrate bonding, readily damage healing, and easy oil sliding, *Science Advances,* 5, 11, eaaw5643.
[18] L. Wang and T. J. McCarthy, Covalently Attached Liquids: Instant Omniphobic Surfaces with Unprecedented Repellency, *Angew Chem Int Ed Engl,* 55, 1, 244-8 (2016).
[19] B. Khatir, S. Shabanian, and K. Golovin, Design and High-Resolution Characterization of Silicon Wafer-like Omniphobic Liquid Layers Applicable to Any Substrate, *ACS Appl Mater Interfaces,* 12, 28, 31933-31939 (2020).
[20] M. Dhar, D. Sarkar, A. Das, S. K. A. Rahaman, D. Ghosh, and U. Manna, 'Rewritable' and 'liquid-specific' recognizable wettability pattern, *Nat Commun,* 15, 1, 5838 (2024).
[21] T. P. Nhung Nguyen, P. Brunet, Y. Coffinier, and R. Boukherroub, Quantitative Testing of Robustness on Superomniphobic Surfaces by Drop Impact, *Langmuir,* 26, 23, 18369-18373 (2010).
[22] H. Zhao, K.-C. Park, and K.-Y. Law, Effect of Surface Texturing on Superoleophobicity, Contact Angle Hysteresis, and “Robustness”, *Langmuir,* 28, 42, 14925-14934 (2012).
[23] A. Tuteja, W. Choi, J. M. Mabry, G. H. McKinley, and R. E. Cohen, Robust omniphobic surfaces, *Proceedings of the National Academy of Sciences,* 105, 47, 18200-18205 (2008).
[24] P. Zhu, T. Kong, X. Tang, and L. Wang, Well-defined porous membranes for robust omniphobic surfaces via microfluidic emulsion templating, *Nat Commun,* 8, 15823 (2017).
[25] S. H. Choi *et al.*, Scalable Multistep Roll-to-Roll Printing of Multifunctional and Robust Reentrant Microcavity Surfaces via a Wetting-Induced Process, *Advanced Materials,* 37, 5, 2411064 (2025).
[26] P. Sun *et al.*, Achieving Extreme Pressure Resistance to Liquids on a Super-Omniphobic Surface with Armored Reentrants, *Small Methods,* 8, 4, 2201602 (2024).
[27] O. Adarraga, C. Agustin-Saenz, I. Bustero, and F. Brusciotti, Superhydrophobic and oleophobic microtextured aluminum surface with long durability under corrosive environment, *Sci Rep,* 13, 1, 1737 (2023).

[28] T. Ma, J. Ma, C. Yang, J. Zhang, and J. Cheng, Robust, Multiresponsive, Superhydrophobic, and Oleophobic Nanocomposites via a Highly Efficient Multifluorination Strategy, *ACS Applied Materials & Interfaces,* 13, 24, 28949-28961 (2021).
[29] T. Nishino, M. Meguro, K. Nakamae, M. Matsushita, and Y. Ueda, The Lowest Surface Free Energy Based on −CF3 Alignment, *Langmuir,* 15, 13, 4321-4323 (1999).
[30] Y. Kawashima and N. Kudo, Toxicity and toxicokinetics of perfluorooctanoic acid in himans and animals, *The Journal of Toxicological Sciences,* 28, 49–57 (2003).
[31] M. Houde *et al.*, Fractionation and bioaccumulation of perfluorooctane sulfonate (PFOS) isomers in a lake Ontario food web, *Environ. Sci. Technol.,* 42, 9397–9403 (2008).
[32] L. Chen, S. Huang, R. H. A. Ras, and X. Tian, Omniphobic liquid-like surfaces, *Nature Reviews Chemistry,* 7, 2, 123-137 (2023).
[33] D. F. Cheng, C. Urata, M. Yagihashi, and A. Hozumi, A statically oleophilic but dynamically oleophobic smooth nonperfluorinated surface, *Angew Chem Int Ed Engl,* 51, 12, 2956-9 (2012).
[34] Z. Li, M. Wei, Y. Zhu, J. Liu, W. Wei, and X. Li, Fabrication of photocurable liquid-like easy-cleaning coatings based on a polydimethylsiloxane-modified silicone resin, *Progress in Organic Coatings,* 187, 108169 (2024).
[35] M. Sun, G. S. Watson, Y. Zheng, J. A. Watson, and A. Liang, Wetting properties on nanostructured surfaces of cicada wings, *J Exp Biol,* 212, 19, 3148-55 (2009).
[36] R. Rakitov and S. N. Gorb, Brochosomal coats turn leafhopper (Insecta, Hemiptera, Cicadellidae) integument to superhydrophobic state, *Proc Biol Sci,* 280, 1752, 20122391 (2013).
[37] A. Lafuma and D. Quéré, Slippery pre-suffused surfaces, *EPL (Europhysics Letters),* 96, 5, (2011).
[38] T. S. Wong *et al.*, Bioinspired self-repairing slippery surfaces with pressure-stable omniphobicity, *Nature,* 477, 7365, 443-7 (2011).
[39] N. R. Geraldi *et al.*, Double-sided slippery liquid-infused porous materials using conformable mesh, *Sci Rep,* 9, 1, 13280 (2019).
[40] G. G. Wells *et al.*, Snap evaporation of droplets on smooth topographies, *Nat Commun,* 9, 1, 1380 (2018).
[41] H. Xu *et al.*, Droplet attraction and coalescence mechanism on textured oil-impregnated surfaces, *Nature Communications,* 14, 1, 4901 (2023).
[42] X. Han, W. Li, H. Zhao, J. Li, X. Tang, and L. Wang, Slippery damper of an overlay for arresting and manipulating droplets on nonwetting surfaces, *Nature Communications,* 12, 1, 3154 (2021).
[43] P. Baumli *et al.*, The challenge of lubricant-replenishment on lubricant-impregnated surfaces, *Adv Colloid Interface Sci,* 287, 102329 (2021).
[44] S. Sett, X. Yan, G. Barac, L. W. Bolton, and N. Miljkovic, Lubricant-Infused Surfaces for Low-Surface-Tension Fluids: Promise versus Reality, *ACS Appl Mater Interfaces,* 9, 41, 36400-36408 (2017).
[45] P. Hao, X. Lou, L. Tang, F. Wang, Z. Chong, and L. Guo, Solvent-free fabrication of slippery coatings from edible raw materials for reducing yogurt adhesion, *Progress in Organic Coatings,* 162, 106590 (2022).

[46] D. J. Sutherland *et al.*, Hemp-Based Sustainable Slippery Surfaces: Icephobic and Antithrombotic Properties, *ACS Sustainable Chemistry & Engineering,* 11, 6, 2397-2403 (2023).
[47] L. P. Xu, D. Han, X. Wu, Q. Zhang, X. Zhang, and S. Wang, A Green Route for Substrate-Independent Oil-Repellent Coatings, *Sci Rep,* 6, 38016 (2016).
[48] A. K. Kota, G. Kwon, W. Choi, J. M. Mabry, and A. Tuteja, Hygro-responsive membranes for effective oil-water separation, *Nat Commun,* 3, 1025 (2012).
[49] Y. A. Lee *et al.*, Slippery, Water-Infused Membrane with Grooved Nanotrichomes for Lubricating-Induced Oil Repellency, *Adv Sci (Weinh),* 9, 13, e2103950 (2022).
[50] H. F. Bohn and W. Federle, Insect aquaplaning: Nepenthes pitcher plants capture prey with the peristome, a fully wettable water-lubricated anisotropic surface, *Proceedings of the National Academy of Sciences,* 101, 39, 14138-14143 (2004).
[51] T. Berninger, N. Dietz, and O. Gonzalez Lopez, Water-soluble polymers in agriculture: xanthan gum as eco-friendly alternative to synthetics, *Microb Biotechnol,* 14, 5, 1881-1896 (2021).
[52] I. Chang, A. K. Prasidhi, J. Im, H.-D. Shin, and G.-C. Cho, Soil treatment using microbial biopolymers for anti-desertification purposes, *Geoderma,* 253-254, 39-47 (2015).
[53] I. Chang *et al.*, Review on biopolymer-based soil treatment (BPST) technology in geotechnical engineering practices, *Transportation Geotechnics,* 24, (2020).
[54] A. Gharsallaoui, B. Rogé, J. Génotelle, and M. Mathlouthi, Relationships between hydration number, water activity and density of aqueous sugar solutions, *Food Chemistry,* 106, 4, 1443-1453 (2008).
[55] S. K. Amit, M. M. Uddin, R. Rahman, S. M. R. Islam, and M. S. Khan, A review on mechanisms and commercial aspects of food preservation and processing, *Agriculture & Food Security,* 6, 1, (2017).
[56] U. Bauer, H. F. Bohn, and W. Federle, Harmless nectar source or deadly trap: Nepenthes pitchers are activated by rain, condensation and nectar, *Proceedings of the Royal Society B: Biological Sciences,* 275, 1632, 259-265 (2007).
[57] U. Bauer and W. Federle, The insect-trapping rim of Nepenthes pitchers: surface structure and function, *Plant Signal Behav,* 4, 11, 1019-23 (2009).
[58] F. Ç. Cebeci, Z. Wu, L. Zhai, R. E. Cohen, and M. F. Rubner, Nanoporosity-Driven Superhydrophilicity: A Means to Create Multifunctional Antifogging Coatings, *Langmuir,* 22, 6, 2856-2862 (2006).
[59] J. D. Smith *et al.*, Droplet mobility on lubricant-impregnated surfaces, *Soft Matter,* 9, 6, 1772-1780 (2013).
[60] Y. Cong, W. Zhang, C. Liu, and F. Huang, Composition and Oil-Water Interfacial Tension Studies in Different Vegetable Oils, *Food Biophysics,* 15, 2, 229-239 (2019).
[61] F. Dawson *et al.*, Self-Assembled, Hierarchical Structured Surfaces for Applications in (Super)hydrophobic Antiviral Coatings, *Langmuir,* 38, 34, 10632-10641 (2022).
[62] A. Jaishankar and G. H. McKinley, A fractional K-BKZ constitutive formulation for describing the nonlinear rheology of multiscale complex fluids, *Journal of Rheology,* 58, 6, 1751-1788 (2014).
[63] S. Armstrong, G. McHale, R. Ledesma-Aguilar, and G. G. Wells, Pinning-Free Evaporation of Sessile Droplets of Water from Solid Surfaces, *Langmuir,* 35, 8, 2989-2996 (2019).
